# The complete mitochondrial genome of *Metapenaeopsis mogiensis* (Decapoda: Penaeidae)

**DOI:** 10.1080/23802359.2018.1535863

**Published:** 2018-11-25

**Authors:** Shengping Zhong, Yanfei Zhao, Qin Zhang

**Affiliations:** Key Laboratory of Marine Biotechnology, Guangxi Institute of Oceanology, Beihai, China

**Keywords:** Mitochondrial genome, *Metapenaeopsis mogiensis*, Decapoda

## Abstract

*Metapenaeopsis* is one of most important genera of Penaeidae. However, the systemically classification and taxonomic studies have so far been limited. In this study, we report the complete mitochondrial genome sequence of *M. mogiensis*. The mitogenome has 15,973 base pairs (66.7% A + T content) and made up of total of 37 genes (13 protein-coding, 22 transfer RNAs and two ribosomal RNAs), and a putative control region. This study was the second available complete mitogenomes of *Metapenaeopsis* and will provide useful genetic information for future phylogenetic and taxonomic classification of *Metapenaeopsis*.

Penaeid shrimp (Decapoda: Penaeidae) is a diverse group of economically important resource in crustacean fisheries (Chan et al. 2008). *Metapenaeopsis* is the most diverse genus within Penaeidae, in which 70 species and six subspecies have been revised recently. *Metapenaeopsis* shrimps are morphologically similar; the shape of the petasma is one of the most important distinguishing taxonomic features, which make identification difficult especially the shape of copulatory organ is similar (Cheng et al. 2015). Moreover, systemically classifications on them have not been done and phylogenetic relationships within them remain largely unknown. The complete mitochondrial genome is useful molecular techniques for systemically classifications. In spite of the wealth of morphological information available for *Metapenaeopsis*, adequate genetic information about the genus is still limited (Kim et al. 2016). Here, we report the second complete mitochondrial genome sequence of genus *Metapenaeopsis*, which will provide a better insight into phylogenetic assessment and taxonomic classification.

Muscle samples of *M. mogiensis* from five individuals were collected from GuangXi province, China (Beihai, 21.353591 N, 109.123656 E), and the whole body specimen (#GQ0193) were deposited at Marine biological Herbarium, Guangxi Institute of Oceanology, Beihai, China. The total genomic DNA was extracted from the muscle of the specimens using an SQ Tissue DNA Kit (OMEGA, Guangzhou, China) following the manufacturer’s protocol. DNA libraries (350 bp insert) were constructed with the TruSeq NanoTM kit (Illumina, San Diego, CA) and were sequenced (2 × 150 bp paired-end) using HiSeq platform at Novogene Company, China. Mitogenome assembly was performed by MITObim (Hahn et al. 2013). The complete mitogenome of *M. dalei* (GenBank accession no. NC_029457) was chosen as the initial reference sequence for MITObim assembly. Gene annotation was performed by MITOS (Bernt et al. 2013).

The complete mitogenome of *M. mogiensis* was 15,973 bp in length (GenBank accession no. MG833230), and containing the typical set of 13 protein-coding, 22 tRNA and two rRNA genes, and a putative control region. The overall base composition of the mitogenome was estimated to be A 33.9%, T 33.7%, C 19.7% and G 12.7%, with a high A + T content of 67.6%, which is similar, but slightly higher than *Melicertus latisulcatus* (66.7%) (Zhong et al. 2018). The gene order in *M. mogiensis* is highly similar to that found in *M. dalei*, which consistent with similar shape of copulatory organ between *M. mogiensis* and *M. dalei*. Moreover, the result of phylogenetic tree of 14 species (including other 13 species from family Penaeidae in NCBI) also supported the close relationship between *M. mogiensis* and *M. dalei* (Figure 1), as they shared the same clade with the highest bootstrap value. The complete mitochondrial genome sequence of *M. mogiensis* was the second sequenced mitogenomes within the genus *Metapenaeopsis*, which will contribute to further evolutionary mitogenome and revisionary taxonomy studies for the genus *Metapenaeopsis*, and related genera.

**Figure 1.**
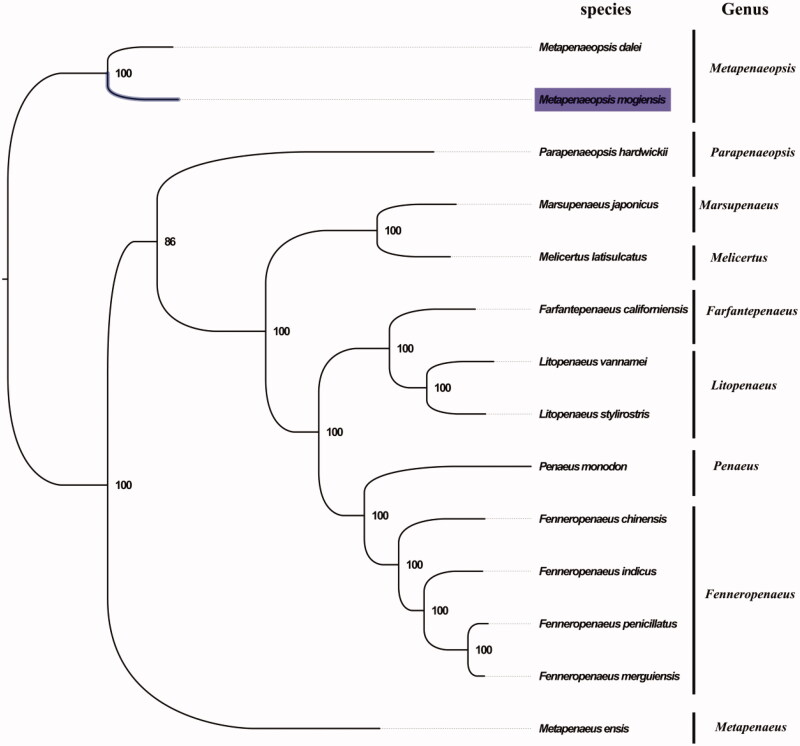
Phylogenetic tree of 14 species in family Penaeidae. The complete mitogenomes is downloaded from GenBank and the phylogenic tree is constructed by maximum-likelihood method with 100 bootstrap replicates. The bootstrap values were labeled at each branch nodes. The gene's accession number for tree construction is listed as follows: *Metapenaeopsis dalei* (NC_029457), *Parapenaeopsis hardwickii* (NC_030277), *Marsupenaeus japonicus* (NC_007010), *Melicertus latisulcatus* (MG821353), *Farfantepenaeus californiensis* (NC_012738), *Litopenaeus vannamei* (NC_009626), *Litopenaeus stylirostris* (NC_012060), *Penaeus monodon* (NC_002184), *Fenneropenaeus chinensis* (NC_009679), *Fenneropenaeus indicus* (NC_031366), *Fenneropenaeus penicillatus* (NC_026885), *Fenneropenaeus merguiensis* (NC_026884), and *Metapenaeus ensis* (NC_026834).
